# PDLIM2 repression by ROS in alveolar macrophages promotes lung tumorigenesis

**DOI:** 10.1172/jci.insight.144394

**Published:** 2021-03-08

**Authors:** Liwen Li, Fan Sun, Lei Han, Xujie Liu, Yadong Xiao, Alyssa D. Gregory, Steven D. Shapiro, Gutian Xiao, Zhaoxia Qu

**Affiliations:** 1 UPMC Hillman Cancer Center, Pittsburgh, Pennsylvania, USA.; 2 Department of Microbiology and Molecular Genetics, University of Pittsburgh School of Medicine, Pittsburgh, Pennsylvania, USA.; 3 Department of Medicine, University of Pittsburgh Medical Center, University of Pittsburgh, Pittsburgh, Pennsylvania, USA.

**Keywords:** Oncology, Cancer, Lung cancer, Macrophages

## Abstract

One of the most fundamental and challenging questions in the field of cancer is how immunity is transformed from tumor immunosurveillance to tumor-promoting inflammation. Here, we identified the tumor suppressor PDZ-LIM domain–containing protein 2 (PDLIM2) as a checkpoint of alveolar macrophages (AMs) important for lung tumor suppression. During lung tumorigenesis, PDLIM2 expression in AMs is downregulated by ROS-activated transcription repressor BTB and CNC homology 1 (BACH1). PDLIM2 downregulation leads to constitutive activation of the transcription factor STAT3, driving AM protumorigenic polarization/activation and differentiation from monocytes attracted from the circulation to suppress cytotoxic T lymphocytes and promote lung cancer. PDLIM2 downregulation also decreases AM phagocytosis. These findings establish ROS/BACH1/PDLIM2/STAT3 as a signaling pathway driving AMs for lung tumor promotion.

## Graphical abstract

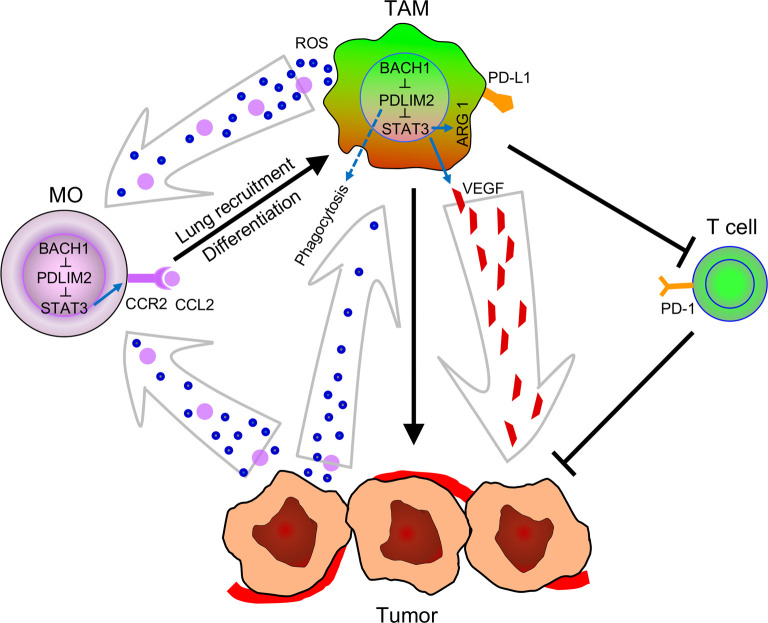

## Introduction

Macrophages are the most abundant immune cells in the lung and serve as key sentinels of the lung, warding off pathogens and maintaining immune and tissue homeostasis (1–4). They are also the main culprits of lung diseases and in particular lung cancer, the leading cause of cancer-related deaths in both men and women (5, 6). Yet, it remains largely unknown how these key immune cells are deregulated to promote lung cancer.

Lung macrophages are highly plastic and heterogeneous, consisting of 2 main subtypes: alveolar macrophages (AMs) and interstitial macrophages (IMs) in the steady-state, healthy lung (1–4). AMs are long-lived, self-renewing cells derived embryonically from hematopoietic stem cells (7) and make up most macrophages in the lung (and are often simply referred to as lung macrophages). They are located in the airspace of the alveoli and express CD11c on the surface. IMs reside within the parenchymal space (interstitium) between adjacent alveoli and express CD11b but not CD11c. Under certain conditions such as lung injury, blood monocytes, which express surface markers most similar to IMs, may be recruited into the lung and differentiate into macrophages (8). However, little is known about the relationship among these lung macrophage populations and their functions, particularly under lung cancer pathogenesis. In fact, it has yet to be examined whether and how monocytes recruited from the circulation are differentiated into lung macrophages, and AMs in particular, during lung tumorigenesis.

Our recent studies have identified STAT3 and NF-κB RelA (also known as p65), 2 master inflammatory transcription factors that also function as proto-oncogenes in lung and many other cancers (9–14), as the intrinsic drivers of lung macrophage protumorigenic activities (15, 16). Whereas their activation in lung macrophages is associated with pulmonary inflammation, tumor progression, and poor survival of patients with lung cancer, deletion of either STAT3 or RelA from lung macrophages in mice represses protumorigenic but boosts antitumorigenic immunity and therefore suppresses lung tumorigenesis. It remains unknown how the tightly regulated STAT3 and RelA become constitutively activated in lung macrophages during lung tumorigenesis.

In this regard, we have demonstrated that through promoting the ubiquitination and proteasomal degradation of nuclear STAT3 and RelA, the PDZ-LIM domain–containing protein PDLIM2 functions as a tumor suppressor particularly important for lung cancer suppression (9, 17–19). PDLIM2, also known as SLIM or Mystique, is expressed most highly in the lung and in particular type II lung epithelial cells and AMs (9, 20–22). We thus hypothesized that PDLIM2 also acts as an inhibitory checkpoint of AM differentiation and protumorigenic function by targeting STAT3, RelA, or both. Moreover, we sought to determine the role of PDLIM2 in AM phagocytosis and its regulation in AMs. Epigenetic repression of PDLIM2 has been shown in tumor cells, but its regulation has not been examined in nonmalignant or immune cells (9, 17–19, 23, 24).

Here, we provide genetic evidence showing that although dispensable for the development and functions of lung macrophages under pathogen-free conditions, PDLIM2 increased AM phagocytosis during lung tumorigenesis. It also restricted the pulmonary recruitment of monocytes and subsequent differentiation into IMs and AMs as well as AM protumorigenic activation via STAT3 repression, thereby relieving cytotoxic T lymphocyte (CTL) suppression and preventing lung cancer. Further, we found that PDLIM2 was decreased in lung macrophages by oxidative stress–activated transcription repressor BTB and CNC homology 1 (BACH1), and that PDLIM2 repression was associated with poor survival of patients with lung cancer. These findings provide mechanistic insights into how the lung maintains immune and tissue homeostasis for its physiological function and how this unique immunosuppressive environment is hijacked for the pathogenesis of lung tumors and other lung diseases associated with oxidative stress.

## Results

### AM-intrinsic PDLIM2 is critical for AM phagocytosis and for restricting the protumorigenic activation and CTL suppression activity of AMs in lung tumorigenesis.

**Figure 1:**
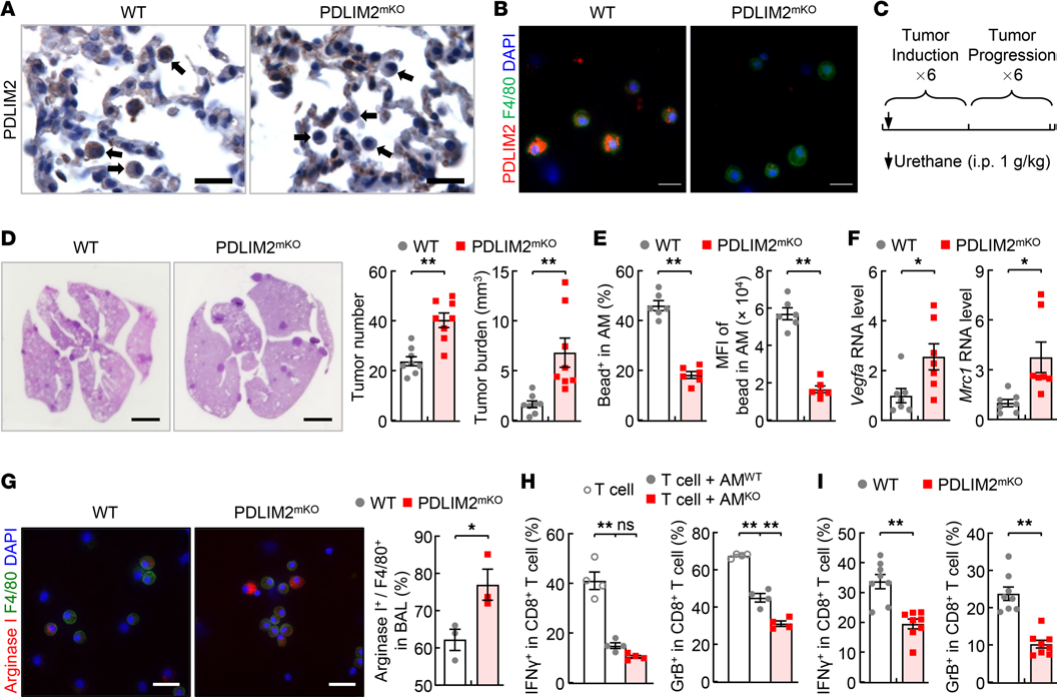
Critical role of cell-intrinsic PDLIM2 in AM phagocytosis and restricting AM protumorigenic activation and suppression of CTLs during lung tumorigenesis. (**A** and **B**) IHC and IF staining showing PDLIM2-selective deletion in pulmonary myeloid cells of PDLIM2^mKO^ mice. Arrows indicate myeloid cells. Scale bar: 20 μm. (**C**) Experimental schedule of lung tumor induction by urethane. (**D**) Tumor examination and H&E staining showing increased lung tumor numbers and tumor burden in urethane-treated PDLIM2^mKO^ mice (*n* = 8). Scale bar: 2.5 mm. (**E**) FACS analysis showing defective phagocytic ability of AMs from urethane-treated PDLIM2^mKO^ mice (*n* = 6). (**F**) qPCR showing increased expression of *Vegfa* and *Mrc1* in the AMs of urethane-treated PDLIM2^mKO^ mice (*n* = 7); 18S rRNA was used as internal control. (**G**) Immunofluorescent (IF) analysis showing increased Arginase 1 in the AMs of urethane-treated PDLIM2^mKO^ mice (*n* = 3). Scale bar: 20 μm. (**H**) Ex vivo coculture assays showing increased repression of CD8^+^ T cells by PDLIM2^-/-^ AMs (*n* = 4). The activity of CD8^+^ T cells was analyzed 2 days after coculture with AMs. (**I**) FACS analysis showing decreased activation of lung CD8^+^ T cells in urethane-treated PDLIM2^mKO^ mice (*n* = 8). Student’s *t* test (2 tailed, unpaired) (**D–G**, and **I**) and ordinary 1-way ANOVA (**H**) were performed, and data represent mean ± SEM. *P < 0.05; **P < 0.01; ns, not statistically significant; GrB, Granzyme B.

We aimed to determine the significance of PDLIM2 in AMs given its tumor suppressor role in lung cancer and its high expression in AMs (9). To this end, we generated PDLIM2^fl/fl^/lysozyme M-Cre mice (PDLIM2^mKO^) in which PDLIM2 was selectively deleted from myeloid cells (Figure 1, A and B). Like PDLIM2^–/–^ mice, under pathogen-free conditions, PDLIM2^mKO^ mice were healthy and showed no apparent abnormalities in the development of myeloid and other immune cells, including lung macrophages (Supplemental Figures 1 and 2; supplemental material available online with this article; https://doi.org/10.1172/jci.insight.144394DS1).

We then tried to examine whether PDLIM2 is involved in AM regulation during lung pathogenesis. To do so, we employed urethane-treated mice, a model of lung cancer relevant to humans, and in particular adenocarcinoma, the most common type of lung cancer that accounts for about 40% of all lung cancers (9–11, 15, 16, 25–27). Urethane treatment induced lung cancers in both WT and PDLIM2^mKO^ mice (Figure 1, C and D). However, PDLIM2^mKO^ mice developed significantly more lung tumors with larger tumor burden compared with WT mice. Interestingly, AMs in urethane-treated PDLIM2^mKO^ mice exhibited a significantly lower phagocytic ability in comparison with those in WT mice under the same treatment (Figure 1E). These data suggest that cell-intrinsic PDLIM2 is required for optimal AM phagocytosis during lung tumorigenesis.

PDLIM2 also controls AM protumorigenic polarization/activation during lung tumorigenesis. In comparison with AMs in urethane-treated WT mice, AMs in PDLIM2^mKO^ mice with the same treatment expressed significantly more arginase, a hallmark of the protumorigenic polarization of macrophages, and significantly higher RNA levels of vascular endothelial growth factor A (*Vegfa*) and mannose receptor C type 1 (*Mrc1*, also known as CD206), 2 other hallmarks of macrophage protumorigenic activation (Figure 1, F and G). Consistently, AMs of PDLIM2^mKO^ mice exhibited ex vivo a higher ability to suppress CTLs, and PDLIM2^mKO^ mice had significantly lower CTL activation in the lung compared with WT mice (Figure 1, H and I). The total numbers of lung CD4^+^ and CD8^+^ T cells as well as CD4^+^ T cell activation and Treg cell differentiation were comparable in those mice (Supplemental Figure 3, A–E). These data suggest that during lung tumorigenesis, cell-intrinsic PDLIM2 restricted lung macrophages and AMs in particular from repressing lung CD8^+^ CTLs for tumor suppression.

### Cell-intrinsic PDLIM2 confines the pulmonary recruitment and macrophage differentiation of monocytes in lung tumorigenesis.

**Figure 2:**
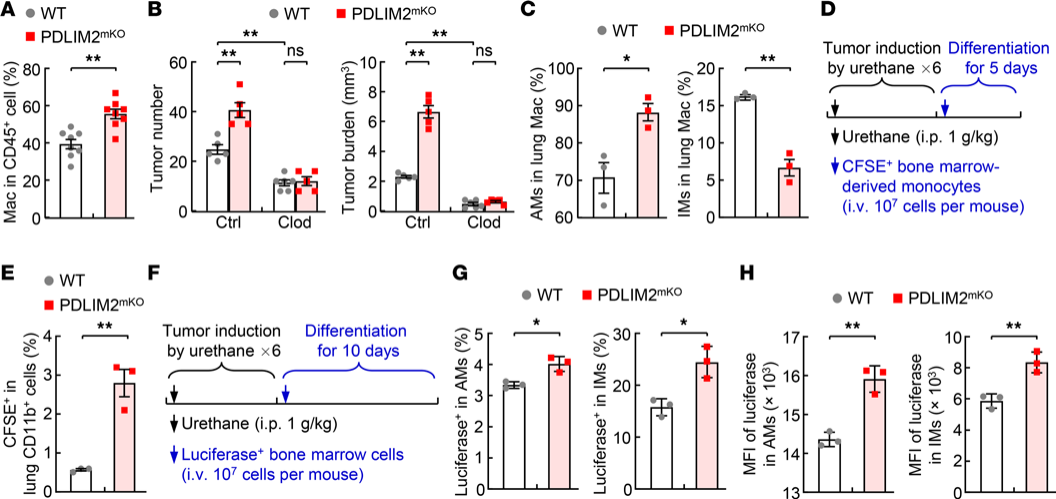
Cell-intrinsic PDLIM2 restriction of lung recruitment and differentiation of bone marrow–derived monocytes into IMs and AMs during lung tumorigenesis. (**A**) FACS analysis showing increased lung macrophages (Mac) in urethane-treated PDLIM2^mKO^ mice (*n* = 8). Absolute numbers of lung macrophages are shown in Supplemental Figure 4A. (**B**) Tumor examination showing better prevention of lung tumorigenesis in urethane-treated PDLIM2^mKO^ mice by clodronate depletion of macrophages and to a comparable level in WT mice (*n* ≥ 5). Ctrl, control; Clod, clodronate. The depletion efficiencies of lung macrophages are shown in Supplemental Figure 4B. (**C**) FACS analysis showing increased percentage of AMs but decreased percentage of IMs among total lung macrophages in urethane-treated PDLIM2^mKO^ mice (*n* = 3). (**D**) Experimental schedule of lung tumor induction and adoptive transfer of CFSE-labeled bone marrow–derived monocytes in WT mice. (**E**) FACS analysis showing increased lung recruitment of the transplanted CFSE-labeled monocytes that were derived from bone marrow cells of PDLIM2^mKO^ mice (*n* = 3). (**F**) Experimental schedule of lung tumor induction and adoptive transfer of luciferase-expressing bone marrow cells in WT mice. (**G** and **H**) FACS analysis showing increased AM and IM differentiation from the transplanted bone marrow cells of PDLIM2^mKO^ mice expressing luciferase (*n* = 3). Student’s *t* test (2 tailed, unpaired) (**A**, **C**, **E**, **G**, and **H**) and ordinary 1-way ANOVA (**B**) were performed, and data represent mean ± SEM. *P < 0.05; **P < 0.01; ns, not statistically significant.

The total numbers of lung neutrophils, DCs, and monocytes, like those of different lymphocytes in the lung, were also comparable in WT and PDLIM2^mKO^ mice treated with urethane (Supplemental Figure 3, A and F). However, there was a significantly greater number of lung macrophages in the PDLIM2^mKO^ mice (Figure 2A and Supplemental Figure 4A). To examine the role of lung macrophages in lung tumorigenesis, in particular in the increased lung tumorigenesis in the PDLIM2^mKO^ mice, we used clodronate to deplete macrophages in vivo as described previously (28). Clodronate depletion of macrophages indeed prevented urethane-induced lung tumors in both WT and PDLIM2^mKO^ mice, as evidenced by the significantly decreased tumor numbers and tumor burdens (Figure 2B and Supplemental Figure 4B). More importantly, both lung tumorigenesis and macrophages in PDLIM2^mKO^ mice were suppressed to levels comparable to those in WT mice. These data indicate that PDLIM2 deficiency in myeloid cells led to an increase of protumor lung macrophages during lung tumorigenesis.

Interestingly, the ratio of AMs in total lung macrophages was increased, whereas the ratio of IMs was decreased in PDLIM2^mKO^ mice (Figure 2C). No differences were observed in proliferation or apoptosis of either AMs or IMs in the WT and PDLIM2^mKO^ mice (Supplemental Figure 4, C and D). These data suggest that the increased macrophages in the lung of PDLIM2^mKO^ mice might be due to increased differentiation of these cells. We thus examined whether PDLIM2^KO^ monocytes show increased pulmonary recruitment and differentiation into IMs and AMs, given that blood monocytes can be recruited into the lung and differentiate into macrophages under certain conditions such as lung injury (8). We used CFSE to label monocytes that were in vitro–derived from the bone marrow cells of urethane-treated WT and PDLIM2^mKO^ mice and compared their pulmonary recruitments in lung tumorigenesis (Figure 2D). Significantly more CFSE-labeled cells were detected in the lung of urethane-treated mice i.v. injected with CFSE-labeled bone marrow–derived monocytes from urethane-treated PDLIM2^mKO^ mice, in comparison with those injected with the same numbers of CFSE-labeled bone marrow–derived monocytes from WT mice treated with urethane (Figure 2E). To further validate those studies, we i.v. injected bone marrow cells of urethane-treated PDLIM2^mKO^ and WT mice, in both of which luciferase expression was driven by lysozyme M-Cre (same as PDLIM2 deletion in PDLIM2^mKO^ mice), into urethane-treated WT mice (Figure 2F). AMs and IMs expressing luciferase were detected in all the mice (Figure 2, G and H). However, mice injected with the cells from PDLIM2^mKO^ mice had significantly more luciferase-expressing AMs and IMs. These data together imply that during lung tumorigenesis, monocytes were recruited from the circulation into the lung to sequentially differentiate into IMs and AMs for lung cancer promotion, and that PDLIM2 restrained this pathogenic process.

### PD-L1/PD-1 blockade suppresses the increased lung tumorigenesis by myeloid PDLIM2 deletion.

**Figure 3:**
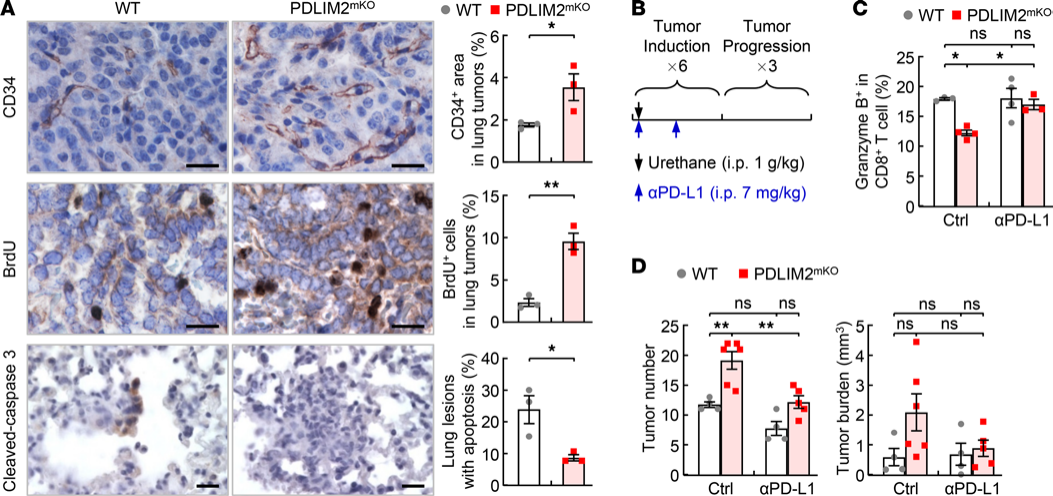
PD-L1/PD-1 blockade suppression of increased lung tumorigenesis by myeloid PDLIM2 deletion. (**A**) IHC staining showing increased tumor angiogenesis and tumor cell proliferation but decreased tumor cell apoptosis in urethane-treated PDLIM2^mKO^ mice (*n* = 3). Scale bar: 20 μm. (**B**) Experimental schedule of lung tumor induction and PD-L1 antibody blockade. (**C**) FACS analysis showing recovery of lung CD8^+^ T cell activation in urethane-treated PDLIM2^mKO^ mice by PD-L1 antibody (*n* ≥ 3). (**D**) Decreased tumor numbers and tumor burden in the lungs of urethane-treated PDLIM2^mKO^ mice by PD-L1 antibody (*n* ≥ 4). Student’s *t* test (2 tailed, unpaired) (**A**) and ordinary 1-way ANOVA (**C** and **D**) were performed, and data represent mean ± SEM. *P < 0.05; **P < 0.01; ns, not statistically significant.

In association with the increased AM differentiation and protumorigenic activation as well as decreased AM phagocytosis and pulmonary CTL activation, the lung tumors in PDLIM2^mKO^ mice had significantly higher angiogenesis and proliferation but decreased apoptosis (Figure 3A). These data demonstrated that PDLIM2 prevented myeloid cells and AMs in particular from promoting lung cancer.

Our recent studies indicated that although its expression is downregulated in most human lung cancers and in our animal models of lung cancer, PD-L1 is expressed on AMs, and in particular, those associated with tumors (25). To validate in vivo the role of PD-L1 inherently expressed on AMs in lung tumorigenesis, we examined whether PD-L1 blockade suppressed the increased lung cancer development in the urethane-treated PDLIM2^mKO^ mice (Figure 3B). PD-L1 blockade indeed reversed the decreased lung CTL activation and the elevated lung tumorigenesis in PDLIM2^mKO^ mice (Figure 3, C and D). Of note, PD-L1 blockade had no significant effect on the urethane-treated WT mice. Altogether, these data suggest that PDLIM2 restrained AM differentiation/expansion, lowering the potential PD-L1/PD-1 interaction between AMs and CTLs and thereby releasing the brake on CTL antitumor activity.

### Myeloid PDLIM2 exerts a lung tumor–suppressive role mainly through targeting STAT3.

**Figure 4:**
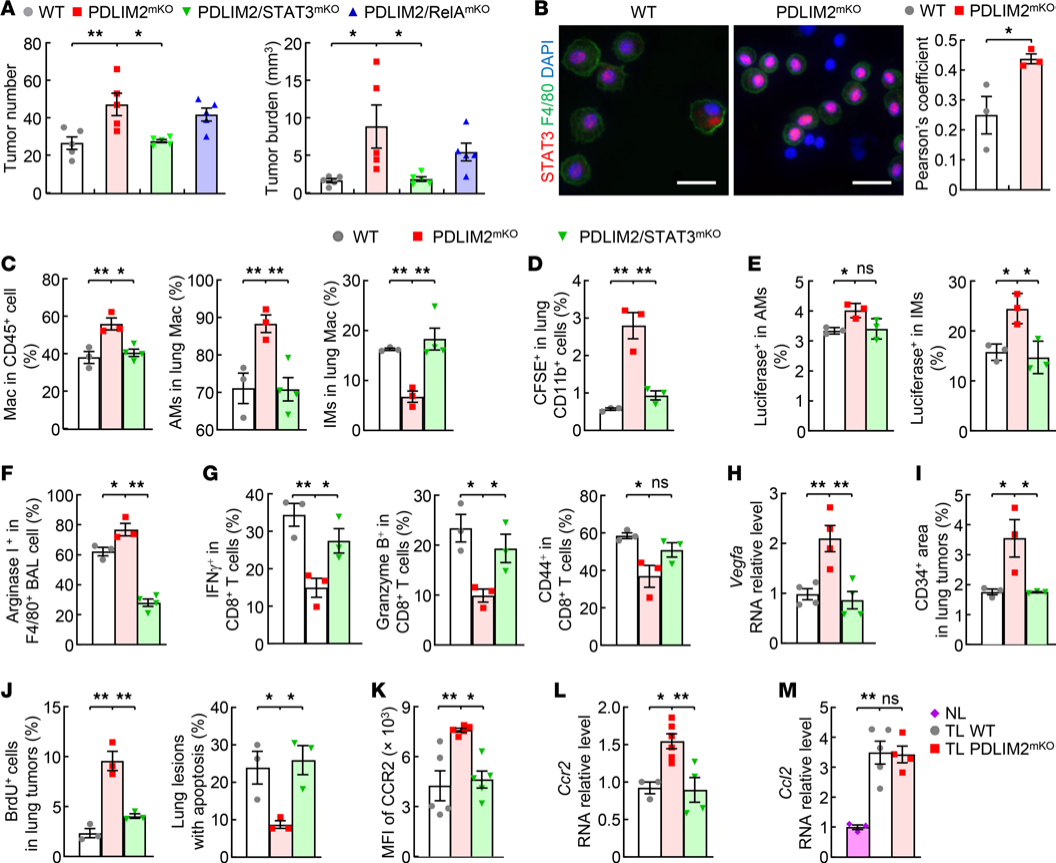
Phenotype reversal in urethane-treated PDLIM2^mKO^ mice by STAT3 codeletion. (**A**) Inhibition of the increased lung tumors in urethane-treated PDLIM2^mKO^ mice by STAT3 but not RelA codeletion (*n* = 5). (**B**) Increased nuclear STAT3 in AMs of urethane-treated PDLIM2^mKO^ mice (IF analysis). Scale bar: 20 μm. Nuclear STAT3 in F4/80^+^ cells was analyzed by ImageJ and represented with Pearson’s correlation coefficient (*n* = 3). (**C**–**E**) STAT3 codeletion inhibited (**C**) the increase of total lung macrophages, the increase of AM ratio, and decrease of IM ratio, and (**D** and **E**) the increased lung recruitment and IM/AM differentiation of bone marrow–derived monocytes in urethane-treated PDLIM2^mKO^ mice (FACS analysis, *n* ≥ 3). (**F**–**L**) In urethane-treated PDLIM2^mKO^ mice, STAT3 codeletion inhibited the increased Arginase 1 in AMs (F, *n* = 3, IF analysis), decreased lung CD8^+^ T cell activation (**G**, *n* = 3, FACS analysis), increased *Vegfa* expression in AMs (**H**, *n* = 4, qPCR analysis), increased lung tumor angiogenesis (I, *n* = 3, IHC CD34 staining), increased proliferation and decreased apoptosis of lung tumor cells (**J**, *n* = 3, IHC assays), increased CCR2 expression on blood monocytes (**K**, *n* = 5, FACS analysis, the gating strategy and representative FACS assays are shown in Supplemental Figure 7), and increased *Ccr2* expression in monocytes derived from bone marrow cells (**L**, *n* ≥ 3, qPCR). (**M**) qPCR showing comparable *Ccl2* increase in lung tissues of urethane-treated WT and PDLIM2^mKO^ mice (*n* ≥ 3; NL, normal lung; TL, tumor-bearing lung). Data shown in **A–D**, and **F–J** are representative of 2 independent experiments with similar results. Ordinary 1-way ANOVA (**A** and **C–M**) and Student’s *t* test (2 tailed, unpaired) (**B**) were performed, and data represent mean ± SEM. *P < 0.05; **P < 0.01; ns, not statistically significant.

To investigate the mechanism underlying the tumor-suppressive role of myeloid-intrinsic PDLIM2 in lung cancer, we simultaneously deleted STAT3 or RelA from myeloid cells in PDLIM2^mKO^ mice because STAT3 and RelA are 2 of the most well-known and best-studied targets of PDLIM2 (17–19, 29–31). In addition, SOCS3, A20 (TNFIAP3), and CYLD, the transcriptional targets of STAT3 and RelA (13, 14), were increased in the lung macrophages of mice with lung cancers (Supplemental Figure 5). STAT3 codeletion completely blocked the increased lung tumorigenesis in PDLIM2^mKO^ mice by urethane, but RelA deletion had no statistically significant effect (Figure 4A), indicating that myeloid PDLIM2 suppresses lung cancer largely through targeting STAT3. In line with this, significantly higher STAT3 activation but comparable RelA activation was detected in AMs from PDLIM2^mKO^ mice compared with those from WT mice, as evidenced by their nuclear expression levels, an activation marker for STAT3 and RelA (Figure 4B and Supplemental Figure 6). This is in contrast to the tumor suppression by cancer cell–intrinsic PDLIM2, which depends on both STAT3 and RelA (9).

STAT3 codeletion in myeloid cells reversed all those changes by PDLIM2 deletion during lung tumorigenesis: elevated pulmonary recruitment of monocytes from the circulation, expanded AM differentiation from IMs and blood monocytes, heightened AM protumorigenic activation, decreased lung CTL activation, increased tumor angiogenesis and tumor cell proliferation, and reduced tumor cell apoptosis (Figure 4, C–J).

To further validate and expand these studies, we tried to define the mechanism by which PDLIM2/STAT3 signaling controls the pulmonary recruitment of blood monocytes, the prerequisite for AM expansion and lung cancer promotion. We examined the expression levels of CCR2 (CD192) in the blood monocytes of WT, PDLIM2^mKO^, and PDLIM2/STAT3^mKO^ mice treated with urethane. CCR2 is a key determinant of monocyte trafficking through binding its ligand monocyte chemoattractant protein-1 (MCP-1/CCL2) (32). Indeed, CCR2 on blood monocytes was significantly increased by PDLIM2 deletion, and the increase was blocked by STAT3 codeletion (Figure 4K and Supplemental Figure 7), and likewise for its expression in bone marrow–derived monocytes (Figure 4L). Of note, *Ccl2* expression was increased in the lung during lung tumorigenesis, but to a similar level in the PDLIM2^mKO^ and WT mice (Figure 4M). These data suggest that PDLIM2 restricted the lung recruitment of monocytes via preventing STAT3 from inducing CCR2 on monocytes, thereby limiting AM differentiation for lung tumor promotion.

### PDLIM2 in AMs is repressed during lung tumorigenesis and PDLIM2 repression is associated with poor survival of patients with lung cancer.

**Figure 5:**
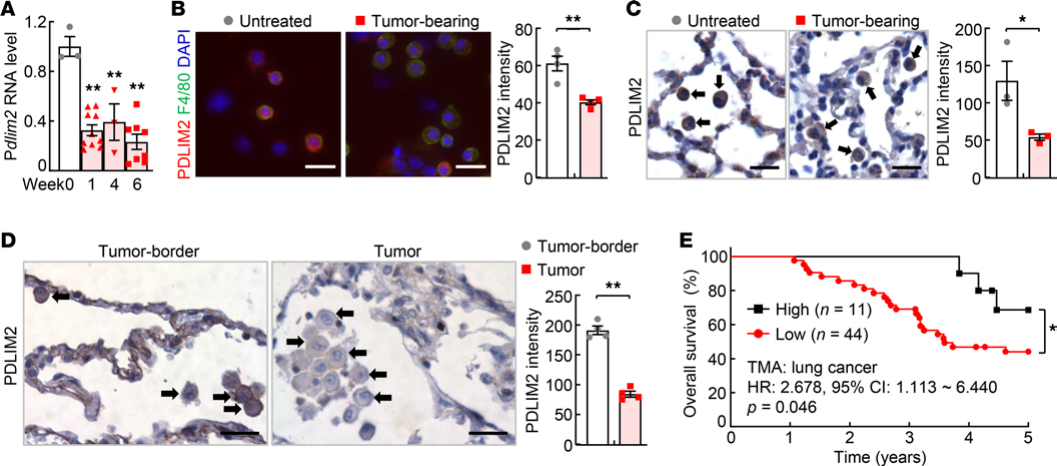
Pathogenic and clinical relevance of PDLIM2 repression in AMs. (**A**) qPCR showing decreased *Pdlim2* expression in BAL cells during lung tumorigenesis (*n* ≥ 3); 18S rRNA was used as internal control. (**B** and **C**) IF and IHC analysis showing decreased PDLIM2 expression in AMs in urethane-treated mice (*n* ≥ 3). Arrows indicate AMs. Scale bar: 20 μm. (**D**) IHC analysis showing decreased PDLIM2 in AMs around human lung cancers compared with those in matched normal human lung tissues (*n* = 4). Arrows indicate AMs. Scale bar: 20 μm. (**E**) Kaplan-Meier survival curve showing a positive association between pulmonary macrophage PDLIM2 expression levels and overall survival of patients with lung cancer in lung cancer tissue microarray. Ordinary 1-way ANOVA (**A**), Student’s *t* test (2 tailed, unpaired) (**B–D**), and Gehan-Breslow-Wilcoxon test (**E**) were performed. Data represent mean ± SEM in **A–D**. *P < 0.05; **P < 0.01.

To investigate the pathogenic and clinical relevance of AM PDLIM2 in lung cancer, we first examined its expression in bronchioalveolar lavage (BAL) cells, of which 90%–95% are AMs (25), from urethane-treated mice. *Pdlim2* expression in BAL cells was significantly decreased 1 week after urethane treatment, and the suppression persisted thereafter (Figure 5A). This was confirmed by immunofluorescent (IF) staining of BAL cells and IHC staining of lung tissues (Figure 5, B and C). Thus, the expression of PDLIM2 was repressed in AMs in the mouse model of lung cancer.

We then validated the mouse studies using clinical samples of patients with lung cancer. PDLIM2 was repressed in tumor-associated macrophages (TAMs)/AMs (Figure 5D and Supplemental Table 1). Of note, the low PDLIM2 expression correlated with poor survival of patients with lung cancer (Figure 5E). PDLIM2 repression in AMs is thus both clinically and pathogenically relevant to lung cancer.

### PDLIM2 downregulation in lung macrophages during lung tumorigenesis is mediated by oxidative stress–activated BACH1.

**Figure 6:**
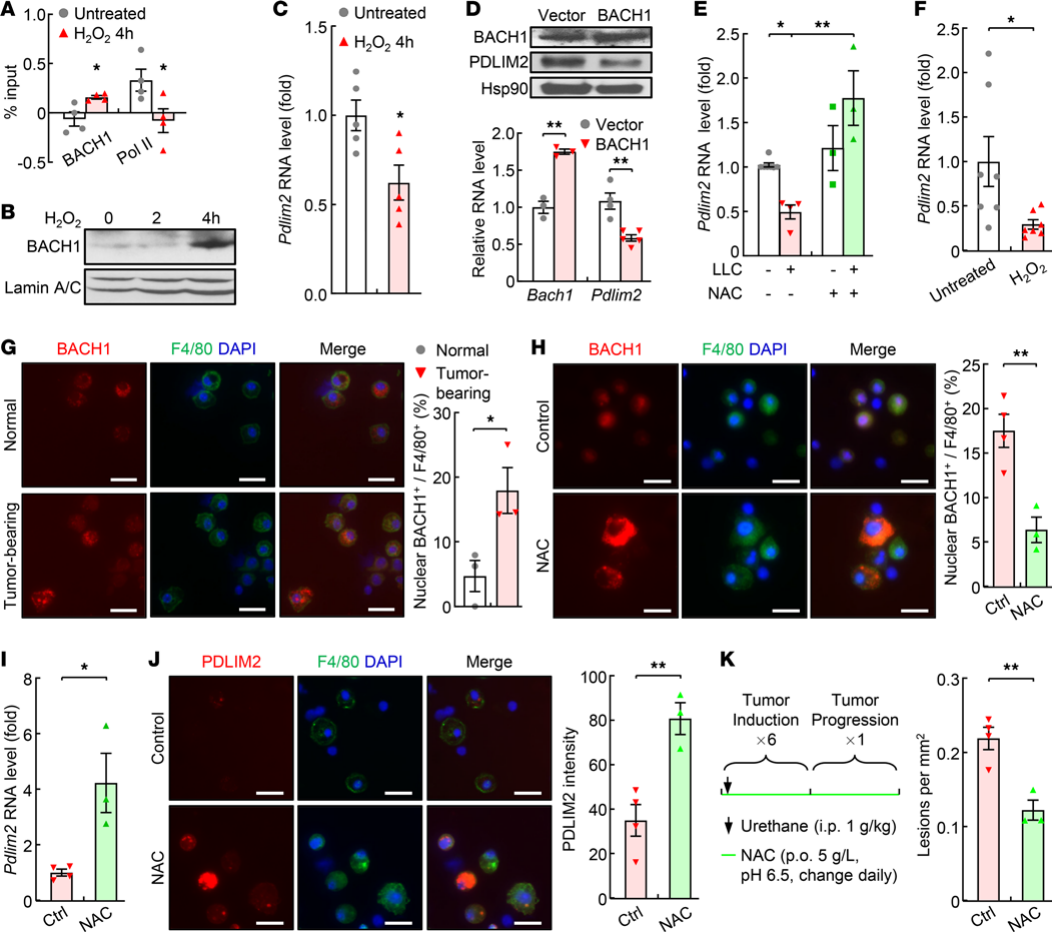
PDLIM2 repression in AMs by ROS-activated BACH1. (**A**) ChIP assays showing more BACH1 but less Pol II bound to the *pdlim2* promoter in RAW264.7 mouse macrophages treated with 500 μM H_2_O_2_ for 4 hours (*n* = 4). (**B**) Nuclear fraction immunoblotting (IB) showing increased nuclear BACH1 in H_2_O_2_-treated RAW264.7 macrophages. (**C**) qPCR showing decreased *Pdlim2* in H_2_O_2_-treated RAW264.7 macrophages (normalized to β-actin, *n* = 5). (**D**) IB and qPCR assays showing decreased PDLIM2 in RAW264.7 cells transfected with BACH1 (*n* ≥ 3). (**E**) qPCR showing decreased *Pdlim2* by Lewis lung carcinoma (LLC) cell coculture but recovery by NAC in RAW264.7 cells (*n* ≥ 3). (**F**) qPCR showing decreased *Pdlim2* in H_2_O_2_-treated primary AMs from mice (*n* = 7). (**G**) IF analysis showing increased nuclear translocation of BACH1 in the AMs of mice with lung tumors (*n* = 3). (**H**) IF analysis showing inhibition of BACH1 nuclear translocation in the AMs of mice with lung tumors by in vivo NAC treatment (*n* ≥ 3). (**I**) qPCR showing increased Pdlim2 in the AMs of mice with lung tumors by in vivo NAC treatment (*n* ≥ 3). (**J**) IF assays showing PDLIM2 induction in the AMs of mice with lung tumors by in vivo NAC treatment (*n* ≥ 3). (**K**) Tumor examination showing NAC prevention of lung tumorigenesis in urethane-treated WT mice (*n* ≥ 3). Experimental schedule of lung tumor induction and in vivo NAC treatment is also shown. Scale bar: 20 μm (**G**, **H**, and **J**). Student’s *t* test (2 tailed, unpaired) (**A**, **C**, **D**, and **F–K**) and ordinary 1-way ANOVA (**E**) were performed, and data represent mean ± SEM in **A** and **C–K**. *P < 0.05; **P < 0.01.

To define the mechanism by which PDLIM2 is repressed in AMs for lung tumor promotion, we analyzed the *pdlim2* promoter and identified a putative BACH1-binding site (Table 1). BACH1 is a transcription repressor of genes involved in the oxidative stress response (33). Of note, oxidative stress is a causative driver of lung diseases and lung cancer in particular (34). ChIP assays detected BACH1 at the BACH1-binding site in H_2_O_2_-treated but not untreated macrophages, which was inversely associated with RNA polymerase II (Pol II) at the *pdlim2* promoter (Figure 6A). Consistently, H_2_O_2_ induced BACH1 nuclear translocation and PDLIM2 downregulation in macrophages (Figure 6, B and C), as did ectopic BACH1 expression (Figure 6D). In contrast, the ROS inhibitor NAC could block the PDLIM2 suppression in macrophages induced by lung tumor cell coculture (Figure 6E). Also, H_2_O_2_ repressed PDLIM2 expression in primary AMs from mice (Figure 6F).

Consistent with the in vitro data, BACH1 was mainly in the nucleus of AMs in mice with lung cancer but in the cytoplasm in untreated mice (Figure 6G). BACH1 nuclear translocation and PDLIM2 repression in AMs in urethane-treated mice could efficiently be blocked by NAC (Figure 6, H–J). Consistent with the prevention of PDLIM2 repression, lung tumorigenesis in the mice was also significantly suppressed (Figure 6K). These data indicate that during lung tumorigenesis, oxidative stress induced BACH1 to enter the nucleus of AMs to bind to the *pdlim2* promoter, thereby repressing PDLIM2 transcription to promote lung cancer.

## Discussion

AMs are the most important guardians that patrol the lung around the clock, and in health, instruct immune tolerance to innocuous inhaled substances but initiate rapid and efficient immune responses to invading pathogens and terminate them after pathogens are cleared to prevent unnecessary inflammation and maintain immune and tissue homeostasis within this essential organ (1–5). Here, we identify the tumor suppressor PDLIM2 as an intrinsic checkpoint of AMs and monocytes for lung cancer suppression.

Similar to its repression in lung precancerous and cancer cells, PDLIM2 downregulation in myeloid cells and in particular AMs is also an important mechanism promoting lung cancer. PDLIM2 downregulation decreases AM phagocytosis while increasing STAT3 activation and promoting AM protumorigenic polarization/activation as well as monocyte pulmonary recruitment and differentiation into AMs to repress CTLs, thereby suppressing both innate and adaptive immunity against lung tumorigenesis. Different from its epigenetic repression in lung and many other cancer cells (9, 13, 17–19, 23, 24), PDLIM2 expression in AMs and monocytes is downregulated by ROS-activated BACH1. Given high ROS production in tumor cells (35), this mechanism may also contribute to PDLIM2 repression in tumor cells. On the other hand, ROS released by tumor cells may contribute to PDLIM2 downregulation in tumor-associated cells, such as TAMs.

In summary, the presented data provide mechanistic insights into lung physiology and lung cancer. Our data also identified PDLIM2 downregulation in AMs and monocytes by ROS-activated BACH1 to increase STAT3 activation as a mechanism driving these immune cells to promote lung cancer. We believe that these insights are applicable to other inflammation-associated diseases because a causal link between oxidative stress and inflammation has been well established in many diseases other than lung cancer and lung diseases.

## Methods

### Animals and lung carcinogenesis.

PDLIM2^fl/fl^ mice, STAT3^fl/fl^ mice, RelA^fl/fl^ mice, and Lysozyme M-Cre mice have been described before (9, 10, 15, 16). Luciferase Cre reporter mice (stock 005125) and FVB/NJ mice (stock 001800) were purchased from The Jackson Laboratory. All mice used were under a pure FVB/NJ background. For lung carcinogenesis, mice were i.p. injected with urethane (1 mg/g body weight, Sigma-Aldrich) once a week for 6 consecutive weeks (9–10, 15, 16, 25, 26). Mice were euthanized for lung inflammation and tumor examinations at 1 week or 6 weeks after urethane treatment. Mice that were also treated with PD-L1 antibodies or N-acetyl-L-cysteine (NAC) were euthanized at 3 weeks or 1 week after urethane treatment, respectively. Surface tumors in mouse lungs were counted by 3 blinded readers under a dissecting microscope, and tumor diameters were measured by microcalipers. For PD-L1 antibody treatment, mice were i.p. injected with PD-L1 or control antibodies (7 μg/g body weight, BioXCell) 2 times per week for 6 consecutive weeks starting at the first day of urethane injection. For NAC treatment, mice were administered NAC in drinking water (5 mg/mL, Sigma-Aldrich) starting at the first day of urethane treatment. Water containing NAC was changed daily and mice were provided the drink ad libitum.

### BAL.

Upon euthanization, mice lungs were lavaged with PBS as described (36). The recovered BAL fluids (BALF) were centrifuged. Pelleted cells from BALF were used for quantitative PCR (qPCR), IF, IHC, and/or flow cytometry (FACS) analysis.

### qPCR analysis.

The indicated tissues or cells were subjected to RNA extraction, RNA reverse transcription, and real-time PCR using TRIzol, reverse transcriptase, and Power SYBR Green PCR Master Mix (Thermo Fisher Scientific) according to the product manufacturer’s protocol (37).

### IF analysis.

Cells were fixed, permeabilized, and subsequently incubated with the indicated primary antibodies, followed by FITC- or TRITC-conjugated secondary antibodies (38, 39). Cells were also counterstained with DAPI for nuclear staining. Stained proteins and their subcellular localizations were detected using a Nikon Eclipse E800 (100 × 1.40 Navil objective) fluorescence microscope and analyzed by ImageJ software (NIH).

### Histology and IHC and human lung tumor tissue microarray assays.

Lung tissues were excised, fixed in formalin, embedded in paraffin, and cut into 4 μm thick sections. Sections were stained with H&E or subjected to sequential incubations with the indicated primary antibodies, biotinylated secondary antibodies, and streptavidin-HRP. Images of the staining were analyzed using ImageJ software (NIH). The human lung tissue microarray has been described before (9, 15, 16). In this assay, macrophages with obvious PDLIM2 staining were scored as 1 or above. Scores were averaged and used for the cutoff of high (≥1) and low (<1) PDLIM2 expression.

### In vivo BrdU labeling.

Mice were i.p. injected with 50 mg/kg BrdU (Sigma-Aldrich) 24 hours prior to euthanization. Mouse lung tissue sections were stained with anti-BrdU (Sigma-Aldrich). More than 500 cells per mouse were counted in randomly selected tumor areas. The BrdU labeling index was calculated as the percentage of labeled cells per total cells counted.

### FACS analysis.

The cells were incubated with the antibodies against cell surface antigens after blocking with αCD16/CD32. The cells were then fixed with paraformaldehyde (2%), permeabilized with saponin (0.5%), and incubated with antibodies against intracellular antigens if needed. For IFN-γ staining, cells were treated with PMA (50 ng/mL), ionomycin (1 μM), brefeldin A (BFA, 3 μg/mL), and monensin (2 μM) for 4 hours before they were stained for FACS analysis. Data were acquired and analyzed by Accuri C6 or LSRFortessa I (BD Biosciences) and FlowJo software (9, 15, 16).

### Peritoneal cell preparation.

Ice-cold PBS was injected into the mouse peritoneal cavity and then recovered from the peritoneal cavity after peritoneum was gently and completely massaged. Peritoneal cells obtained were used for FACS analysis.

### In vitro differentiation of bone marrow–derived monocytes.

Bone marrow cells were flushed from femurs of the indicated mice and cultured for 5 days with 20 ng/mL macrophage colony-stimulating factor (M-CSF) in an ultralow attachment plate (Corning Inc.). Adherent cells were bone marrow–derived macrophages; nonattached cells were used for isolation of bone marrow–derived monocytes with a monocyte isolation kit (Miltenyi Biotec).

### In vivo pulmonary recruitment and AM differentiation of monocytes during lung tumorigenesis.

WT mice treated with urethane (1 mg/g body weight, Sigma-Aldrich, i.p. injection 2 times per week for 6 consecutive weeks) were i.v. injected with (10^7^ cells/mouse) CFSE-labeled monocytes in vitro differentiated from bone marrow cells of the indicated mice treated with urethane or (10^7^ cells/mouse) bone marrow cells from the indicated luciferase-expressing mice. Mice injected with bone marrow–derived monocytes and bone marrow cells were euthanized at 5 days or 10 days, respectively, after cell injection, and the lung tissues were then subjected to FACS to detect CFSE^+^ CD11b^+^ cells or luciferase-expressing AMs and IMs.

### In vitro CTL suppression by AMs.

Splenic CD3^+^ T cells from WT mice were cocultured with PDLIM2-deficient or WT AMs for 2 days followed by FACS analysis to detect IFN-γ^+^ and granzyme B (GrB)^+^ CD8^+^ T cells.

### Ex vivo phagocytosis assays.

Macrophages from fresh mouse lung tissues of the indicated mice were seeded in an ultralow attachment plate (Corning Inc.) for 20 minutes, and then Latex Beads-Rabbit IgG-FITC Complex (Cayman Chemical, 1:100) was directly added and cultured for 2 hours. The phagocytic abilities of AMs were determined by FACS.

### ChIP assays.

Cells were collected after formaldehyde treatment. The chromatin DNA was extracted, broken into fragments of 300–1000 bp in length by sonication, and immunoprecipitated with the indicated antibodies (40). DNA in the IP product was amplified by PCR.

### Subcellular fractionation and immunoblotting assays.

Whole-cell extracts were prepared by lysing cells in RIPA buffer (50 mM Tris-HCl, pH 7.4, 150 mM NaCl, 1 mM EDTA, 0.25% [wt/vol] Na-deoxycholate, 1% [vol/vol] NP-40, 1 mM DTT) (41). Nuclear extracts were prepared by lysing pellets in insoluble nuclear buffer (20 mM Tris, pH 8.0, 150 mM NaCl, 1% [wt/vol] SDS, 1% [vol/vol] NP-40, and 10 mM iodoacetamide) after the cytoplasm was extracted with the hypotonic buffer (20 mM HEPES, pH 8.0, 10 mM KCl, 1 mM MgCl_2_, 0.1% [vol/vol] Triton X-100, and 20% [vol/vol] glycerol) (42). The purity of the nuclear fractions was confirmed by checking Hsp90 (cytoplasm) or lamin B/C (nuclear fraction). All the lysis buffers were supplemented with 1 mM PMSF and a protease inhibitor cocktail (Roche Molecular Biochemicals). The cell extracts were used for immunoblotting assays (43). Briefly, the cell extracts were separated on polyacrylamide gels followed by electrotransfer onto nitrocellulose membranes. After blocking nonspecific protein binding with 5% dry milk, the membranes were sequentially incubated with appropriate primary and HRP-conjugated secondary antibodies and extensively washed with PBS with 0.1% Tween 20 (PBST) after each of the incubation steps. Specific immune complexes were detected by ECL as specified by the manufacturer (Western Lightning ECL Pro; Amersham).

### Antibodies and primers.

Antibodies used for IF, histology, ChIP, FACS, immunoblotting, and in vivo blocking assays, including the company names, catalogue numbers, and dilutions, are listed in Supplemental Table 2. Primers for ChIP and qPCR are listed in Supplemental Table 3.

### Statistics.

Measurements were taken from distinct samples. Student’s *t* test (2 tailed, unpaired) was used to assess significance of differences between 2 groups. Ordinary 1-way ANOVA was used to assess significance of differences among groups of more than 2. Gehan-Breslow-Wilcoxon test was used to compare overall patient survival between high and low macrophage PDLIM2 expression groups. The survival analysis was justified with cancer stage, and demographic information, including sex, age, and smoking statuses of the patients with lung cancer, using Fisher’s exact test or χ^2^ test. All bars in figures represent mean ± SEM. *P* values less than 0.05 and 0.01 were considered statistically significant and highly statistically significant, respectively.

### Study approval.

We have complied with all relevant ethical regulations for animal testing and research. The animal experiments were performed in accordance with the *Guide for the Care and Use of Laboratory Animals* (National Academies Press, 2011). All animals were maintained under pathogen-free conditions and used according to protocols approved by the IACUC of the University of Pittsburgh.

## Author contributions

ZQ and GX conceived and designed the study, led and contributed to all aspects of the analysis, and wrote the manuscript. LL performed the experimental assays related to the urethane model of lung cancer, in vitro differentiation and *Ccr2* RNA expression of bone marrow–derived monocytes, in vivo lung recruitment of CFSE-labeled bone marrow–derived monocytes, in vitro cell line assays on PDLIM2 repression by ROS/BACH1, as well as PDLIM2 downregulation and activation of STAT3 and RelA and BACH1 in AMs in mice harboring lung tumors. FS performed ex vivo phagocytosis assays, FACS assays on CCR2 expression, in vivo analysis of the effect of macrophage depletion on lung tumor genesis and immunology, in vivo NAC treatment experiments, AM differentiation assays using luciferase-expressing bone marrow cells, human lung tumor tissue array staining, and ex vivo assays on H_2_O_2_ repression of PDLIM2 in mouse primary AMs. LH contributed to the experimental assays involving the urethane model of lung cancer, computational predication of BACH1-binding motif within the *pdlim2* promoter, and quantitation of AM/TAM PDLIM2 expression in the human lung tumor tissue array. XL contributed to IF assays of PDLIM2 expression in mouse AMs. LL, FS, and YX contributed to mouse clone maintenance. ADG and SDS provided advice and constructive feedback and edited the manuscript.

## Supplementary Material

Supplemental data

## Figures and Tables

**Table 1:**
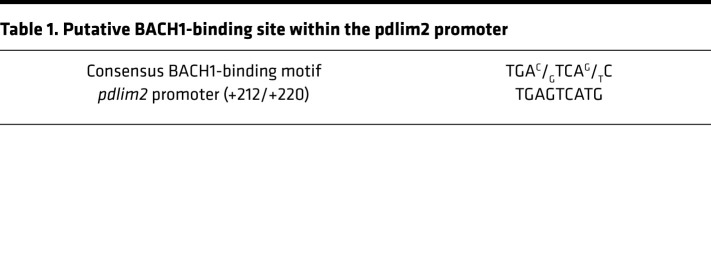
Putative BACH1-binding site within the pdlim2 promoter

## Version 1 02/04/2021

In-Press Preview

## Version 2 03/08/2021

Electronic publication
