# Supplementary material for: PDLIM2 repression by ROS in alveolar macrophages promotes lung tumorigenesis
Source: JCI Insight. 2021 Mar 8;6(5):e144394. doi: 10.1172/jci.insight.144394 (PMC8021114; doi:10.1172/jci.insight.144394)
Supplement: Supplemental data [file jciinsight-6-144394-s199.pdf]

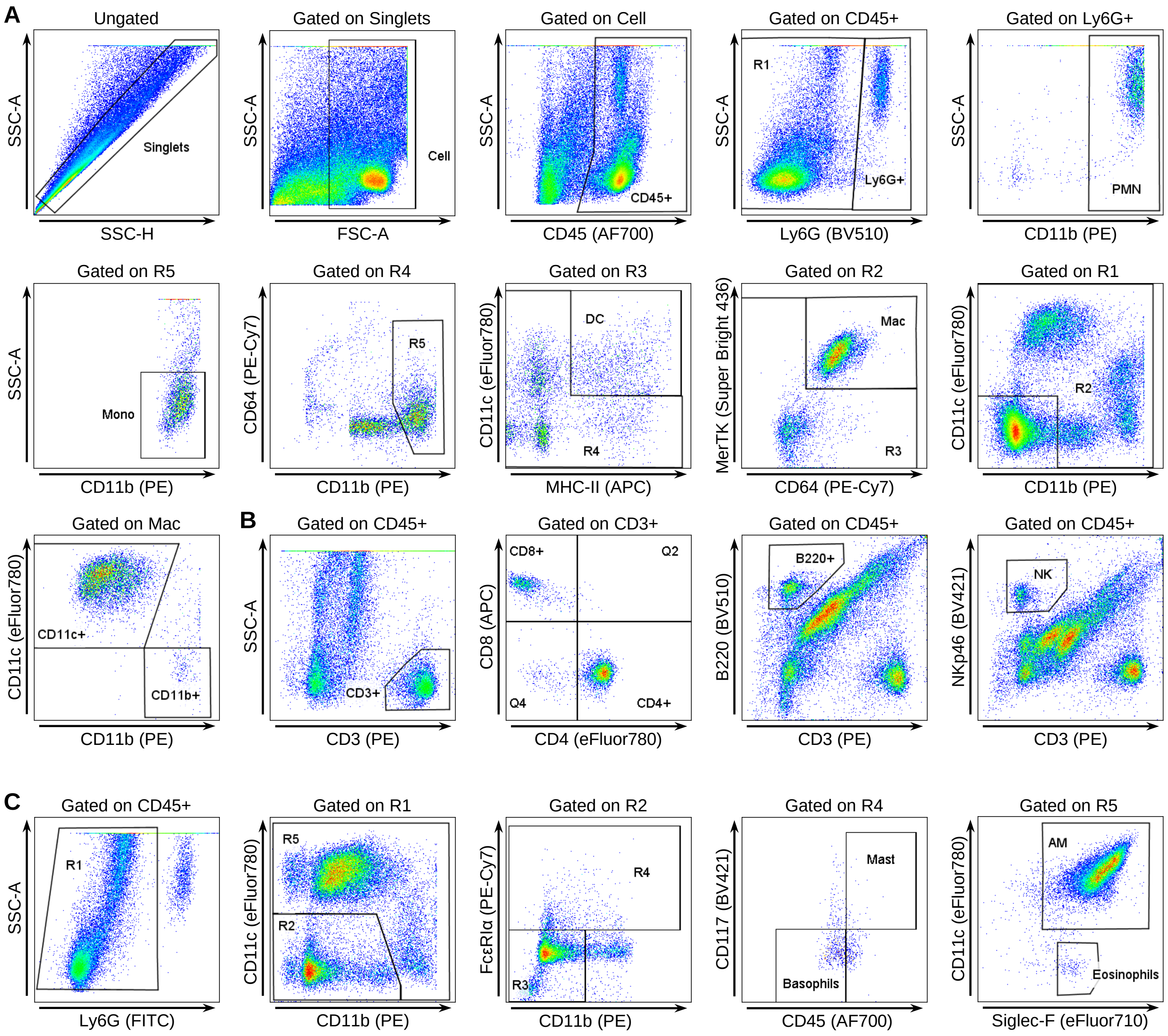


**Supplemental Figure 1. FACS gating strategy for immune cells in normal lung tissue of mice.** (**A**) Gating strategy for neutrophils (PMN), dendritic cells (DC), monocytes (Mono), macrophages (Mac) and their sub-types AMs (CD11c+) and IMs (CD11b+). (**B**) Gating strategy for $CD4^{+}$ T cells (CD4+), $CD8^{+}$ T cells (CD8+), B cells (B220+) and natural killer (NK) cells. (**C**) Gating strategy for basophils, mast cells (Mast) and eosinophils.

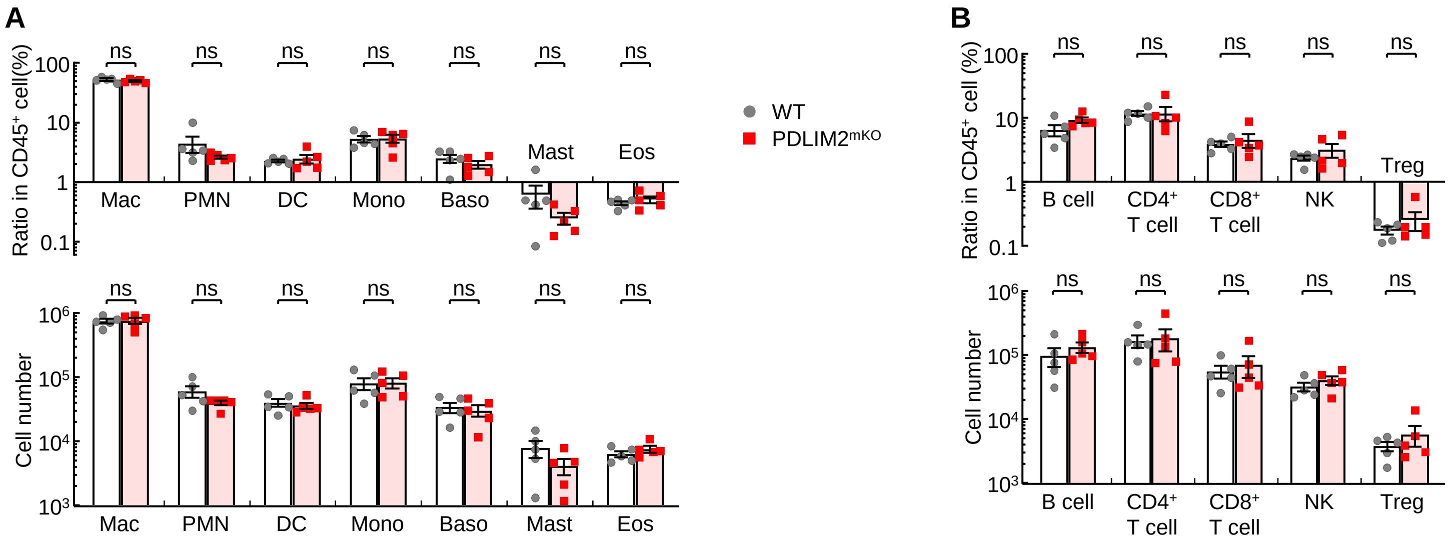


**Supplemental Figure 2. No obvious effect of myeloid PDLIM2 deletion on lung immune cells under normal conditions.** FACS assays showing no significant difference between WT and PDLIM2$^{mKO}$ mice in their lung myeloid (**A**) and lymphocyte (**B**) populations (*n* = 5). Student's *t* test was performed (two tailed, unpaired) and data represent means ± SEM. ns, not statistically significant.

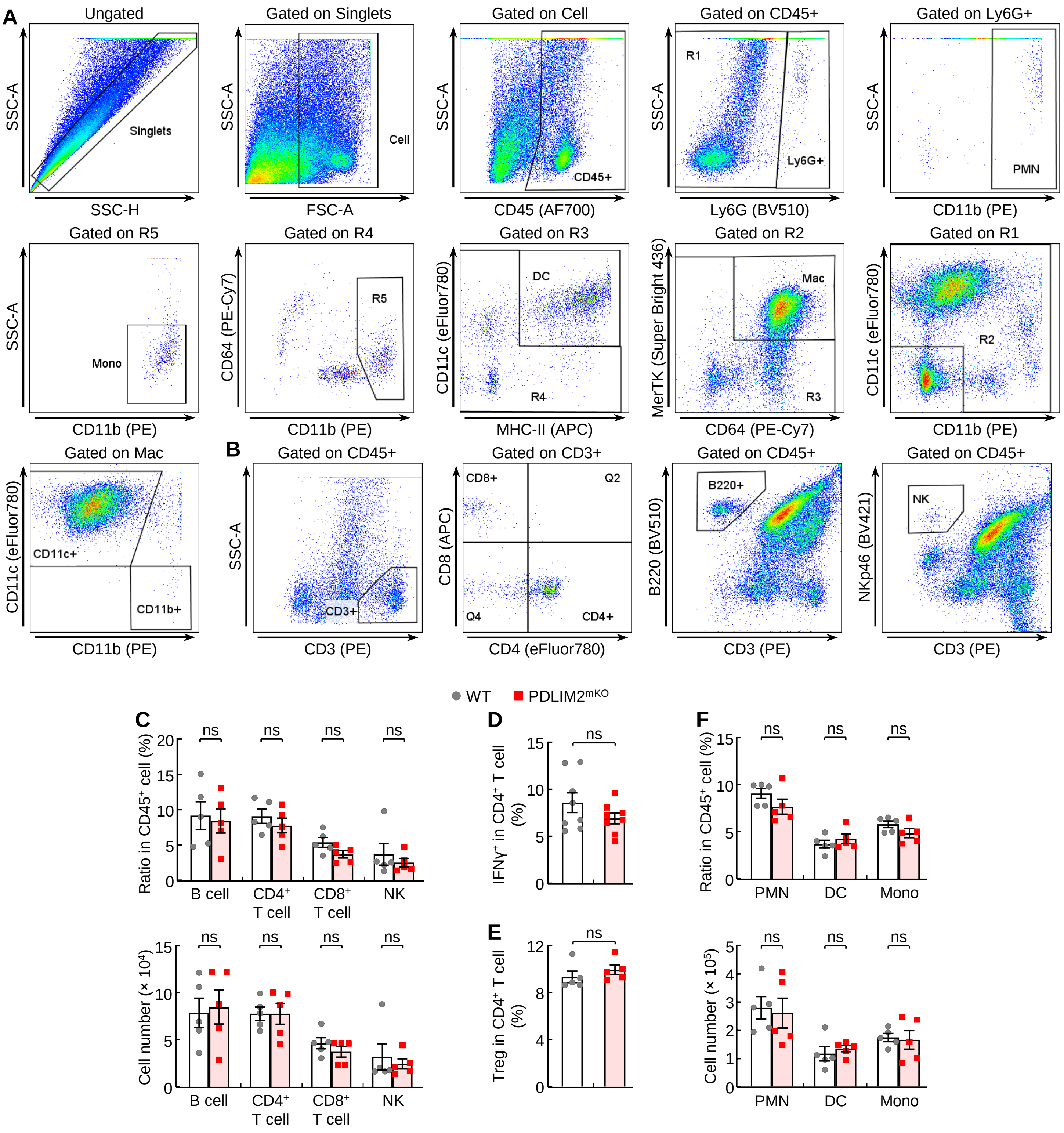

**Supplemental Figure 3. Lung immune profiles of WT and PDLIM2$^{mKO}$ mice treated with urethane.** (**A** and **B**) FACS gating strategy for (A) myeloid cells and (B) lymphoid cells in the lung of urethane-treated mice. PMN, neutrophil; Mac, macrophage; CD11c+, AM; CD11b+, IM; DC, dendritic cells; Mono, monocytes; NK, natural killer cells. (**C**) FACS assays showing no significant difference between urethane-treated WT and PDLIM2$^{mKO}$ mice in their lung lymphocyte populations ($n$ = 5). (**D** and **E**) FACS analysis showing comparable CD4$^{+}$ T-cell activation (E, $n$ = 8) and Treg differentiation (F, $n$ = 5) in the lung of WT and PDLIM2$^{mKO}$ mice treated with urethane. (**F**) FACS assays showing no significant difference between urethane-treated WT and PDLIM2$^{mKO}$ mice in the indicated myeloid cell types ($n$ = 5). Student's $t$ test (two tailed, unpaired) was performed, and data represent means ± SEM in (C-F). ns, not statistically significant.

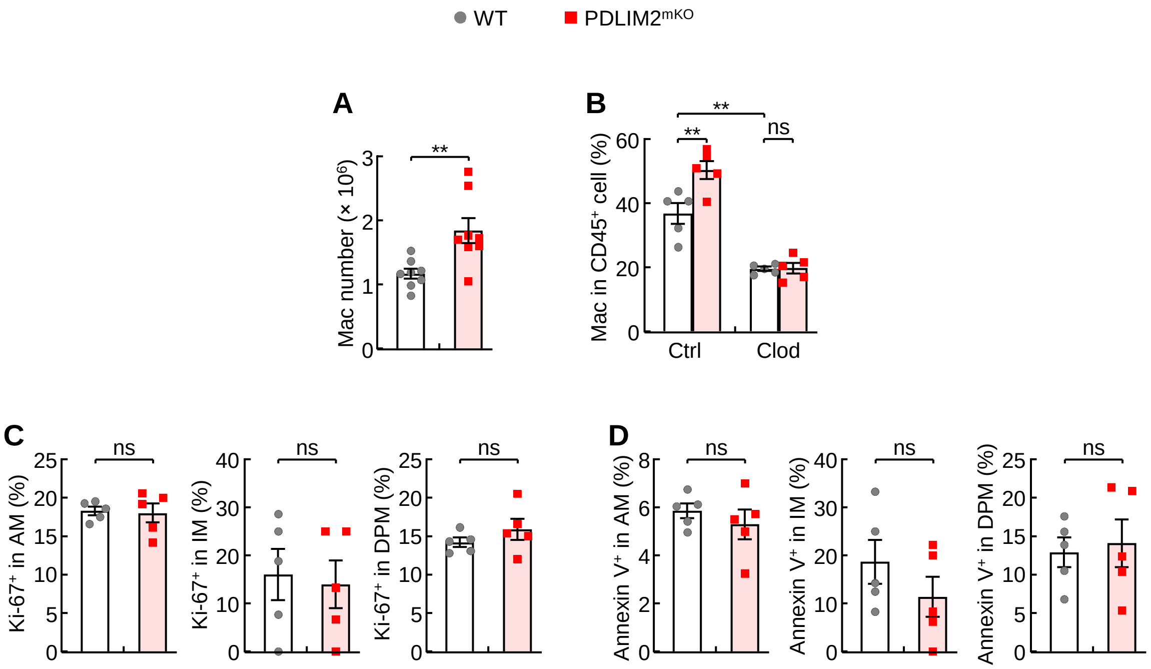


**Supplemental Figure 4. Increased number but not proliferation or apoptosis of lung macrophages in WT and PDLIM2$^{mKO}$ mice treated with urethane.** (**A**) FACS assays showing more macrophages in the lung of urethane-treated PDLIM2$^{mKO}$ mice ($n$ = 8). (**B**) FACS assays showing clodronate (clod) depletion of macrophages in the lung of urethane-treated WT and PDLIM2$^{mKO}$ mice ($n$ = 5). (**C** and **D**) FACS analysis showing comparable proliferation (C) and apoptosis (D) of AMs in the lung of WT and PDLIM2$^{mKO}$ mice treated with urethane ($n$ = 5). Student's $t$ test (two tailed, unpaired) (A, C-D) and Ordinary one-way ANOVA (B) were performed, and data represent means ± SEM. *, $P < 0.05$; **, $P < 0.01$; ns, not statistically significant.

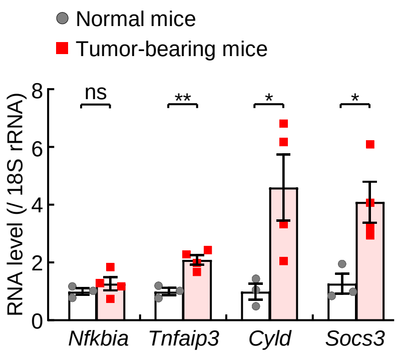


**Supplemental Figure 5. Increased expression of NF-κB and STAT3 inhibitors in lung macrophages in the mice harboring lung cancers.** qPCR analysis showing increased RNA expression of the indicated genes in the lung macrophages of mice treated with urethane for 6 weeks, compared to untreated mice. Student's *t* test was performed (two tailed, unpaired) and data represent means ± SEM ($n \geq 3$). *, $P < 0.05$; **, $P < 0.01$; ns, not statistically significant.

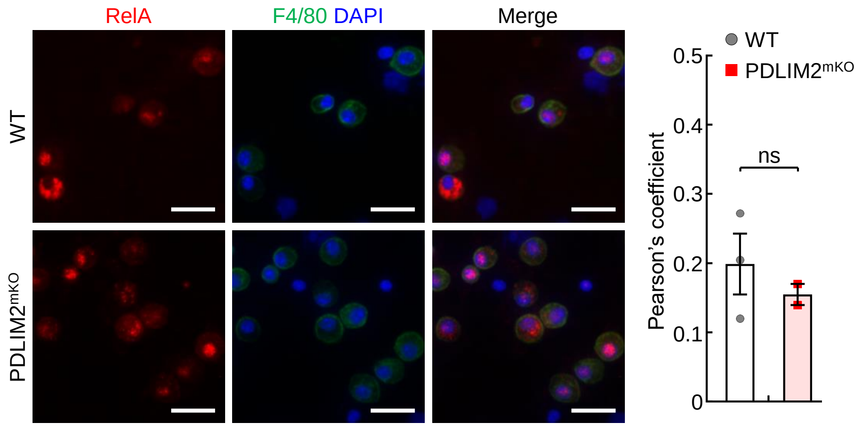


**Supplemental Figure 6. No significant effect of cell-intrinsic PDLIM2 deletion on RelA activation in the lung macrophages of mice with lung tumorigenesis.** IF co-staining of RelA (red) and F4/80 (green) in BAL cells showing no effect of PDLIM2 deletion on RelA nuclear expression in the lung macrophages of urethane-treated mice. Scale bar: 20 µm. The nuclear expression of RelA in F4/80-positive cells was analyzed by image J and represented by Pearson's correlation coefficient. Student's *t* test was performed (two tailed, unpaired) and data represent means ± SEM ($n \geq 2$ mice, 3 images per mouse). ns, not statistically significant.

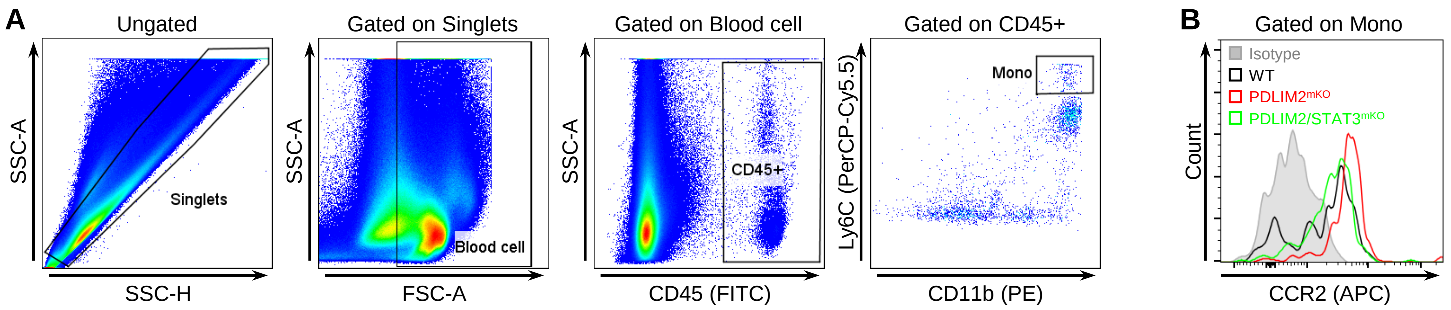


**Supplemental Figure 7. Increase of CCR2 expression on blood monocytes by cell-intrinsic PDLIM2 deletion and inhibition of CCR2 increase by STAT3 co-deletion.** (**A**) FACS gating strategy for blood monocytes. (**B**) Representative FACS assays of CCR2 expression on blood monocytes in the indicated mice treated with urethane.

**Supplemental Table 1. Macrohpage PDLIM2 expression and clinicopathological characteristics of lung cancer patients from the TMA cohort**

| Lung cancer patient cohort | Macrophage PDLIM2 expression level | | | |
|---|---|---|---|---|
| Clinicopathological characteristics | Low (n=47) | High (n=11) | Total (n=58) | *p* -value |
| Sex | | | | 0.5172 |
| Male | 27 | 5 | 32 | |
| Female | 20 | 6 | 26 | |
| Age (mean=65.71 yrs) | | | | 0.3248 |
| < mean | 21 | 7 | 28 | |
| > mean | 26 | 4 | 30 | |
| Smoke | | | | 0.3623 |
| Never | 4 | 1 | 5 | |
| Former | 29 | 9 | 38 | |
| Smoker | 14 | 1 | 15 | |
| T stage | | | | 0.275 |
| T1 | 16 | 7 | 23 | |
| T2 | 25 | 4 | 29 | |
| T3 | 1 | 0 | 1 | |
| T4 | 5 | 0 | 5 | |
| N stage | | | | 0.5833 |
| N0 | 29 | 9 | 38 | |
| N1 | 6 | 1 | 7 | |
| N2 | 8 | 1 | 9 | |
| NX | 4 | 0 | 4 | |
| Clinical stage | | | | 0.3811 |
| 1 | 26 | 9 | 35 | |
| 2 | 5 | 1 | 6 | |
| 3 | 12 | 1 | 13 | |
| N/A | 4 | 0 | 4 | |

## Supplementary Table 2. Antibodies Used

| Antibody | Clone | Catalog number | Company | Usage | Purpose |
|---|---|---|---|---|---|
| Anti-CD45 Alexa Fluor 700 | 30-F11, Rat IgG2b, κ | 103128 | Biolegend, San Diego, CA, USA | 0.5 µl per sample | FACS |
| Anti-CD117 BV421 | ACK2, Rat IgG2b, κ | 135124 | Biolegend, San Diego, CA, USA | 5.0 µl per sample | FACS |
| Anti-NKp46 BV421 | 29A1.4, Rat IgG2a, κ | 137612 | Biolegend, San Diego, CA, USA | 2.5 µl per sample | FACS |
| Anti-B220 BV510 | RA3-6B2, Rat IgG2a, κ | 103248 | Biolegend, San Diego, CA, USA | 2.5 µl per sample | FACS |
| Anti-Ly6G BV510 | 1A8, Rat IgG2a, κ | 127633 | Biolegend, San Diego, CA, USA | 2.5 µl per sample | FACS |
| Anti-Ly6C BV650 | HK1.4, Rat IgG2c, κ | 128049 | Biolegend, San Diego, CA, USA | 1.25 µl per sample | FACS |
| Anti-CD64 PE-Cy7 | X54-5/7.1, Mouse IgG1, κ | 139314 | Biolegend, San Diego, CA, USA | 2.5 µl per sample | FACS |
| Anti-FcεRIα PE-Cy7 | MAR-1, Armenian Hamster IgG | 134318 | Biolegend, San Diego, CA, USA | 2.5 µl per sample | FACS |
| Anti-CCR2 APC | SA203G11, Rat IgG2b, κ | 150627 | Biolegend, San Diego, CA, USA | 2.5 µl per sample | FACS |
| Anti-CD45 FITC | 30-F11, Rat IgG2b, κ | 103107 | Biolegend, San Diego, CA, USA | 0.5 µl per sample | FACS |
| Anti-MerTK Super Bright 436 | DS5MMER, Rat IgG2a, κ | 62-5751-82 | Thermo Fisher Scientific, Waltham,MA, USA | 5.0 µl per sample | FACS |
| Anti-Ki-67 PerCP-eFluor710 | SolA15, Rat IgG2a, κ | 46-5698-82 | Thermo Fisher Scientific, Waltham,MA, USA | 0.3 µl per sample | FACS |
| Anti-PD-1 PerCP-eFluor710 | J43, Armenian hamster IgG | 46-9985-82 | Thermo Fisher Scientific, Waltham,MA, USA | 0.625 µl per sample | FACS |
| Anti-MHC-II APC | M5/114.15.2, Rat IgG2b, κ | 17-5321-82 | Thermo Fisher Scientific, Waltham,MA, USA | 0.15 µl per sample | FACS |
| Anti-CD11c APC-eFluor780 | N418, Armenian hamster IgG | 47-0114-82 | Thermo Fisher Scientific, Waltham,MA, USA | 2.5 µl per sample | FACS |
| Anti-CD3e APC-eFluor780 | 145-2C11, Armenian hamster IgG | 47-0031-82 | Thermo Fisher Scientific, Waltham,MA, USA | 5 µl per sample | FACS |
| Anti-CD4 APC-eFluor780 | RM4-5, Rat IgG2a, κ | 47-0042-82 | Thermo Fisher Scientific, Waltham,MA, USA | 0.625 µl per sample | FACS |
| Anti-Annexin V | VAA-33, Rat IgG2a, κ | BMS147FI | Thermo Fisher Scientific, Waltham,MA, USA | 5 µl per sample | FACS |
| Anti-CD11b PE | M1/70, Rat IgG2b, κ | 12-0112-82 | Thermo Fisher Scientific, Waltham,MA, USA | 0.625 µl per sample | FACS |
| Anti-CD11c PE-Cy7 | N418, Armenian hamster IgG | 25-0114-82 | Thermo Fisher Scientific, Waltham,MA, USA | 2.5 µl per sample | FACS |
| Anti-CD16/CD32 | 93, Rat IgG2a, λ | 14-0161-85 | Thermo Fisher Scientific, Waltham,MA, USA | 1.0 µl per sample | FACS |
| Anti-CD25 PE | PC61.5, Rat IgG1, λ | 12-0251-81 | Thermo Fisher Scientific, Waltham,MA, USA | 0.625 µl per sample | FACS |
| Anti-CD3 PE | 145-2C11, Armenian hamster IgG | 12-0031-83 | Thermo Fisher Scientific, Waltham,MA, USA | 2.5 µl per sample | FACS |
| Anti-CD4 PE-Cy7 | RM4-5, Rat IgG2a, κ | 25-0042-81 | Thermo Fisher Scientific, Waltham,MA, USA | 1.25 µl per sample | FACS |
| Anti-CD44 PE-Cy7 | IM7, Rat IgG2b, κ | 25-0441-81 | Thermo Fisher Scientific, Waltham,MA, USA | 0.625 µl per sample | FACS |
| Anti-CD8 APC | 53-6.7, Rat IgG2a, κ | 17-0081-83 | Thermo Fisher Scientific, Waltham,MA, USA | 0.625 µl per sample | FACS |
| Anti-F4/80 APC | BM8, Rat IgG2a, κ | 17-4801-82 | Thermo Fisher Scientific, Waltham,MA, USA | 2.0 µl per sample | FACS |
| Anti-Foxp3 FITC | FJK-16s, Rat IgG2a, κ | 11-5773-82 | Thermo Fisher Scientific, Waltham,MA, USA | 2.0 µl per sample | FACS |
| Anti-GranzB FITC | NGZB, Rat IgG2a, κ | 11-8898-80 | Thermo Fisher Scientific, Waltham,MA, USA | 0.25 µl per sample | FACS |
| Anti-IFNγ FITC | XMG1.2, Rat IgG1, κ | 11-7311-81 | Thermo Fisher Scientific, Waltham,MA, USA | 1.0 µl per sample | FACS |
| Anti-Ly6C PerCP/Cyanine5.5 | HK1.4, Rat IgG2c, κ | 45-5932-82 | Thermo Fisher Scientific, Waltham,MA, USA | 5.0 µl per sample | FACS |
| Anti-Ly6G FITC | 1A8-Ly6g, Rat IgG2a, κ | 11-9668-82 | Thermo Fisher Scientific, Waltham,MA, USA | 1.0 µl per sample | FACS |
| Anti-Siglec-F PerCP-eFluor710 | 1RNM44N, Rat IgG2a, κ | 46-1702-82 | Thermo Fisher Scientific, Waltham,MA, USA | 0.625 µl per sample | FACS |
| Anti-Luciferase FITC | Luci 21 1-107, Mouse IgG1, κ | NB600-307F | Novus Biologicals, Littleton, CO, USA | 1.0 µl per sample | FACS |
| Rat IgG2a kappa Isotype Control, PE-Cyanine7 | eBR2a | 25-4321-82 | Thermo Fisher Scientific, Waltham,MA, USA | 0.625 µl per sample | FACS |
| Rat IgG2a kappa Isotype Control, Super Bright 780 | eBR2a | 78-4321-82 | Thermo Fisher Scientific, Waltham,MA, USA | 0.3 µl per sample | FACS |
| Armenian Hamster IgG Isotype Control, PerCP-eFluor 710 | eBio299Arm | 46-4888-82 | Thermo Fisher Scientific, Waltham,MA, USA | 0.625 µl per sample | FACS |
| Armenian Hamster IgG Isotype Control, eFluor 450 | eBio299Arm | 48-4888-82 | Thermo Fisher Scientific, Waltham,MA, USA | 2.5 µl per sample | FACS |
| Armenian Hamster IgG Isotype Control, BV510 | HTK888 | 400942 | Biolegend, San Diego, CA, USA | 2.5 µl per sample | FACS |
| Rat IgG2a, κ Isotype Control, BV650 | RTK2758 | 400542 | Biolegend, San Diego, CA, USA | 1.25 µl per sample | FACS |
| Rat IgG2a, κ Isotype Control, PerCP/Cyanine5.5 | RTK2758 | 400531 | Biolegend, San Diego, CA, USA | 1.25 µl per sample | FACS |
| Rat IgG2b, κ Isotype Control, APC | RTK4530 | 400611 | Biolegend, San Diego, CA, USA | 2.5 µl per sample | FACS |

| | | | | | |
|---|---|---|---|---|---|
| Latex Beads-Rabbit IgG-FITC Complex | | 500290 | Cayman Chemical, Ann Arbor, MI, USA | 1:100 | FACS |
| Anti-PD-L1 | 10F.9G2, Rat IgG2b, κ | BE0101 | BioXcell, West Lebanon, NH, USA | 20 μg (*in vitro*); 7 μg/g (*in vivo*) | Blockade |
| Anti-Pol II | 4H8, Mouse IgG1 | 2629 | Cell Signaling Technology, Danvers,MA, USA | 1:50 | ChIP |
| Anti-BACH1 | C-20, goat polyclonal IgG | sc-14700 | Santa Cruz Biotechnology, Dallas, TX,USA | 1:500 (IF); 1:1000 (IB); 1:50 (ChIP) | IF/IB/ChIP |
| Anti-Arginase | N-20, goat polyclonal IgG | sc-18351 | Santa Cruz Biotechnology, Dallas, TX,USA | 1:500 | IF |
| Anti-STAT3 | 79D7, Rabbit IgG | 4904 | Cell Signaling Technology, Danvers,MA, USA | 1:500 | IF |
| Anti-RelA | D14E12, Rabbit IgG | 8242 | Cell Signaling Technology, Danvers,MA, USA | 1:500 | IF |
| Anti-F4/80 | Cl:A3-1, Rat IgG2b | MCA497G | Bio-Rad, Hercules, CA, USA | 1:200 | IF |
| Anti-mouse PDLIM2 | Goat antibidy | EB11878 | Everest Biotech, Ramona, CA, USA | 1:200 (IF); 1:1000 (IB); 1:400 (IHC) | IF/IB/IHC |
| Anti-human PDLIM2 | Rabbit polyclonal antibody | HPA003880 | Sigma-Aldrich, St. Louis,MO, USA | 1:250 | IHC |
| Anti-BrdU | BU-33, monoclonal | B2531 | Sigma-Aldrich, St. Louis,MO, USA | 1:500 | IHC |
| Anti-CD34 | C-18, goat polyclonal IgG | sc-7045 | Santa Cruz Biotechnology, Dallas, TX,USA | 1:500 | IHC |
| Anti-Cleaved Caspase 3 | Asp175 | 9661 | Cell Signaling Technology, Danvers,MA, USA | 1:200 | IHC |
| Anti-Hsp90 | F-8, mouse monoclonal IgG2a | sc-13119 | Santa Cruz Biotechnology, Dallas, TX,USA | 1:1000 | IB |
| Anti-Lamin A/C | 346, mouse monoclonal IgM | sc-7293 | Santa Cruz Biotechnology, Dallas, TX,USA | 1:1000 | IB |
| Donkey anti-Goat TRITC | | A16004 | Thermo Fisher Scientific, Waltham,MA, USA | 1:200 | IF |
| Donkey anti-Rat FITC | | DKXRT-003-DFITC | ImmunoReagents Inc., Raleigh, NC, USA | 1:200 | IF |
| Donkey anti-Rabbit IgG-R | | sc-2095 | Santa Cruz Biotechnology, Dallas, TX,USA | 1:200 | IF |
| Rabbit anti-goat IgG Biotinylated | | sc-2774 | Santa Cruz Biotechnology, Dallas, TX,USA | 1:200 | IHC |
| Goat anti-rabbit IgG Biotinylated | | E0432 | Dako, Carpinteria, CA, USA | 1:200 | IHC |
| Anti-mouse IgG Biotinylated | | BMK-2202 | Dako, Carpinteria, CA, USA | 1:200 | IHC |
| Goat anti-Mouse HRP | | sc-2055 | Santa Cruz Biotechnology, Dallas, TX,USA | 1:5000 | IB |
| Donkey anti-Goat HRP | | sc-2020 | Santa Cruz Biotechnology, Dallas, TX,USA | 1:5000 | IB |

## Supplemental Table 3. Primers Used

| Gene | Species | Accession number | Forward (5' to 3') | Reverse (5' to 3') | Usage |
|---|---|---|---|---|---|
| *18S RNA* | mouse | NR_003278.3 | AGGAATTGACGGAAGGGCAC | GGACATCTAAGGGCATCACA | RT-PCR |
| *Pdlim2* | mouse | NM_145978.2 | GCAGCGTCAACATCTCGAACC | TGCTTCTCGCAGTACAACTCATT | RT-PCR |
| *Vegfa* | mouse | NM_009505.4 | GCACTGGACCCTGGCTTTAC | GTCTCAATCGGACGGCAGTA | RT-PCR |
| *Mrc1* | mouse | NM_008625.2 | TTCAGCAACAGCAAGGCGAA | ATTGAATGCTTGGCGCTGGA | RT-PCR |
| *Ccr2* | mouse | NM_009915.2 | GCCATCATAAAGGAGCCATACC | TGTGGTGAATCCAATGCCCT | RT-PCR |
| *Ccl2* | mouse | NM_011333.3 | GCTGTTCACAGTTGCCGGCTG | GGGCGTTAACTGCATCTGGCT | RT-PCR |
| *Bach1* | mouse | NM_007520.2 | TCACCTGACCGCCGCTTG | ATTGAGGCTGAGCAAGACGTT | RT-PCR |
| *Nfkbia* | mouse | NM_010907.2 | CTGGACTCCATGAAGGACGA | GTGGATGATTGCCAAGTGCAG | RT-PCR |
| *Tnfaip3* | mouse | NM_001166402.1 | GTGACCCTGAAGGACAGTGG | TTGATCAGGTGAGTCGTGCC | RT-PCR |
| *Cyld* | mouse | NM_001128170.2 | CAGGTAGCAGGTTCGGCTG | ACTGGCAAAAAGGAGCCACT | RT-PCR |
| *Socs3* | mouse | NM_007707.3 | CAAGGCCGGAGATTTCGCTT | GGGAAACTTGCTGTGGGTGA | RT-PCR |
| *Pdlim2* | mouse | NC_000080.6 | CCAGGTATAGCTCTGGGGGA | GGGCCAGCTCTAAGCACTAC | ChIP PCR |
